# Risk Factors for Early Urethral Stricture After Mono-Polar Transurethral Prostate Resection: A Single-Center Experience

**DOI:** 10.7759/cureus.19663

**Published:** 2021-11-17

**Authors:** Ahmet Gür, Gökhan Sönmez, Türev Demirtaş, Şevket T Tombul, Kemal Halitgil, Abdullah Demirtaş

**Affiliations:** 1 Urology, Kayseri City Hospital, Kayseri, TUR; 2 History of Medicine and Ethics, Erciyes University, Kayseri, TUR; 3 Urology, Erciyes University, Kayseri, TUR

**Keywords:** stricture, urethral, resection, prostate, transurethral

## Abstract

Aim

This study aimed to investigate the incidence of urethral stricture during the early period after transurethral resection of the prostate (TURP) and the risk factors affecting the development of urethral stricture in patients treated in our clinic.

Material and methods

This retrospective study included patients who underwent TURP due to benign prostate hyperplasia (BPH) and had complete postoperative follow-up data of at least 12 months. Univariate and multivariate logistic regression analyses were performed to evaluate the relationship between urethral stricture and eight parameters (age, body mass index [BMI], prostate volume, number of comorbidities, amount of tissue removed, operative time, perioperative blood loss, and catheterization duration).

Results

Of the 3069 patients who underwent TURP in our clinic during the study period, 1740 patients with complete clinical data were included in the study. Mean age was 67.83 ± 5.80 years and mean body mass index (BMI) was 27.63 ± 4.31 kg/m^2^. Median preoperative prostate volume was 50.0 (range, 41.0-62.0) mm^3^ and the average amount of tissue removed during surgery was 20.0 (range, 12.0-30.0) g. Urethral stricture was detected in 3.9% (67/1740) of the patients during a minimum of 12 months of follow-up period after TURP. In multivariate analysis, prolonged operative time and high comorbidity burden were found to be risk factors for urethral stricture (*p*<0.001 for both).

Conclusion

Early urethral stricture remains an important complication of TURP. Our results show that prolonged operative time and high comorbidity burden are factors that increase the risk of urethral stricture.

## Introduction

Benign prostate hyperplasia (BPH) is a common benign disease, particularly in elderly men [1]. Despite the increasing popularity of laser enucleation techniques in the treatment of BPH, transurethral resection of the prostate (TURP) is a leading surgical technique [2]. TURP continues to attract the attention of urologists, particularly due to its minimal invasiveness compared to open surgeries as well as its highly effective outcomes and low complication rates [3].

In addition to the well-known complications of TURP such as prolonged postoperative bleeding, TURP syndrome, urinary retention, and postoperative urinary infections, urethral stricture remains a leading long-term complication of TURP. In the literature, a wide range of urethral stricture rates after transurethral surgeries has been reported (1.7-9.8%) [4-6].

Although the etiology of urethral stricture after endoscopic urethral surgeries remains unclear, patient-related factors, iatrogenic traumas developing during the surgery, and infections are the most frequently implicated factors. In addition, there are limited data in the literature suggesting that prolongation of the time spent in the urethra during surgery may impair urethral blood flow and cause ureteral epithelial disorders [7-9].

The aim of this study was to evaluate patients who underwent TURP and to investigate the demographic and clinical factors affecting the development of urethral stricture.

## Materials and methods

Study design and patient selection

Of the 3069 patients with at least 12 months' follow-up who underwent TURP due to BPH in Erciyes University Medical School Urology Clinic between January 2009 and November 2020, 1740 patients with complete clinical data were included in this retrospective study.

Other inclusion criteria were as follows: (i) aged 45-75 years, no history of endoscopic urological intervention, (ii) no known history of urethral catheterization, (iii) no history of urethral catheterization or endoscopic urological intervention for any reason after TURP, and (iv) no history of stone removal or urogenital trauma after TURP. Additionally, patients detected with urethral stricture during TURP surgery, patients with intraoperative iatrogenic urethral injury, and patients with a known history of urethral stricture were excluded from the study. In our clinic, TURP surgeries are routinely performed using a 26 Fr resectoscope and a monopolar cautery system as standard. Moreover, at the end of the surgery, a 20 Fr urethral catheter is applied to the patients. Patients who were considered ineligible for the surgical treatment depending on these criteria were also excluded from the study.

Patients were divided into those who did not develop urethral stricture after TURP (Group I, n=1673) and those who developed urethral stricture (Group II, n=67).

Data collection

The hospital database and patient follow-up files were retrospectively analyzed for demographic and clinical characteristics of patients, including age, body mass index (BMI), comorbidities, perioperative blood loss, prostate volume, operative time, amount of tissue removed during TURP, urethral catheterization time, and time to the development of urethral stricture (if any).

Body mass index (BMI) was calculated using the kg/m^2^ formula. Prostate volumes of the patients were retrieved from preoperative imaging reports (ultrasound, computed tomography). Comorbidities included coronary artery disease (CAD), diabetes mellitus (DM), chronic obstructive pulmonary disease (COPD), hypertension, and asthma, which were scored from 0 to 5. Operative time was defined as the time (minutes) from the insertion of the resectoscope into the urethra to the insertion of the catheter. The amount of tissue removed during TURP was defined as the weight of the removed prostate specimen (grams) measured on a precision scale after filtering the fluid of the specimen. Perioperative blood loss was defined as the change in the patient’s pre and postoperative hemoglobin levels (gr/dL).

Patients who had a suspicious urethral stricture in uroflowmetry within the first 12 months after TURP and whose diagnosis was confirmed by cystourethroscopy were accepted as having urethral stricture.

Statistical analysis

Data were analyzed using SPSS for Windows version 20.0 (IBM Corp. Released 2011. IBM SPSS Statistics for Windows, Version 20.0. Armonk, NY: IBM Corp.). The normal distribution of data was determined using the Shapiro-Wilk test and histogram plots. Continuous variables with normal distribution were expressed as mean ± standard deviation (SD) and continuous variables with nonnormal distribution were expressed as median (1^st^-3^rd^ quartile). Categorical variables were presented as frequencies (%). Qualitative data with normal distribution were compared using paired samples t-test. Variables with nonnormal distribution were compared using the Mann-Whitney U test and categorical variables were compared using the chi-square test (Fisher’s exact test and Pearson’s chi-square test). Initially, univariate logistic regression analysis was performed to evaluate the relationship between the eight parameters and urethral stricture. Subsequently, multivariate regression analysis was performed for the parameters associated with urethral stricture in univariate analysis. A p-value of <0.05 was considered significant.

Ethical concern

The study was approved by Erciyes University Medical School Clinical Research Ethics Committee (Approval No: 2021/975). Written and verbal consent were obtained from each participant.

## Results

The mean age was 67.83 ± 5.80 years and the mean BMI was 27.63 ± 4.31 kg/m^2^. Median preoperative prostate volume was 50.0 (range, 41.0-62.0) mm^3^ and the average amount of tissue removed during surgery was 20.0 (range, 12.0-30.0) g. In the study population, urethral stricture was detected in 3.9% (67/1740) patients during a minimum 12-month follow-up period after TURP.

A significant difference was found between the two groups with regard to total prostate volume (p=0.025), the number of comorbidities (p<0.001), and the amount of tissue removed during surgery (p<0.001) (Table 1).

**Table 1:** Demographic and clinical characteristics of the patients BMI: body mass index, COPD: chronic obstructive pulmonary disease Continuous variables with normal distribution were expressed as mean ± standard deviation (SD) and continuous variables with nonnormal distribution were expressed as median (1st-3rd quartile).

Variable	Group 1 (n=1673)	Group 2 (n=67)	p	Overall (n=1740)
Age (years)	67.88 ± 5.75	66.64 ± 6.75	0.144	67.83 ± 5.80
BMI (kg/m^2^)	27.65 ± 4.31	27.06 ± 4.36	0.704	27.63 ± 4.31
Median follow-up period (months)	56.0 (33.0-92.0)	54.0 (36.0-88.0)	0.889	56.0 (34.0-92.0)
Comorbidities (n)	1.0 (0-1.0)	3.0 (2.0-4.0)	<0.001	1.0 (0-2.0)
Prostate volume (mm^3^)	50.0 (41.0-61.0)	55.0 (42.0-75.0)	0.025	50.0 (41.0-62.0)
Operative time (min)	35.0 (25.0-50.0)	60.0 (35.0-65.0)	<0.001	35.0 (25.0-50.0)
Tissue removed (gr)	20.0 (12.0-30.0)	25.0 (12.0-40.0)	0.109	20.0 (12.0-30.0)
Perioperative blood loss (g/dL)	1.0 (0.4-1.4)	1.0 (0.5-2.1)	0.089	1.0 (0.4-1.5)
Postoperative catheterization period (days)	2.83 ± 1.14	3.00 ± 1.05	0.231	2.84 ± 1.14

In univariate and multivariate binary logistic regression analyses, the number of comorbidities (p<0.001) and the length of time spent in the urethra (p<0.001) were found to be effective factors in the development of urethral stricture after TURP, whereas age, BMI, prostate volume, the amount of tissue removed, perioperative blood loss, and postoperative urethral catheterization times did not have a significant effect on the development of urethral stricture (Table 2).

**Table 2:** Binary logistic regression analysis results and relationship between the clinical factors and urethral stricture BMI: body mass index, OR: odds ratio, CI: confidence interval

	Univariate			Multivariate		
	p	OR	95% Cl OR	p	OR	95% CI OR
Age	0.088	0.96	[0.929, 1.005]	-	-	-
BMI	0.267	0.97	[0.913, 1.026]	-	-	-
Prostate volume	0.029	1.01	[1.001, 1.021]	0.337	1.01	[0.990, 1.028]
Number of comorbidities	<0.001	2.03	[1.743, 2.362]	<0.001	2.00	[1.705-2.355]
Amount of tissue removed	0.008	1.02	[1.005, 1.031]	0.065	0.97	[0.942, 1.002]
Operative time	<0.001	1.04	[1.029, 1.054]	<0.001	1.05	[1.032-1.067]
Perioperative blood loss	0.012	1.29	[1.058, 1.579]	0.127	1.19	[0.952, 1.485]
Catheterization period	0.230	1.13	[0.924, 1.387]	-	-	-

**Figure 1:**
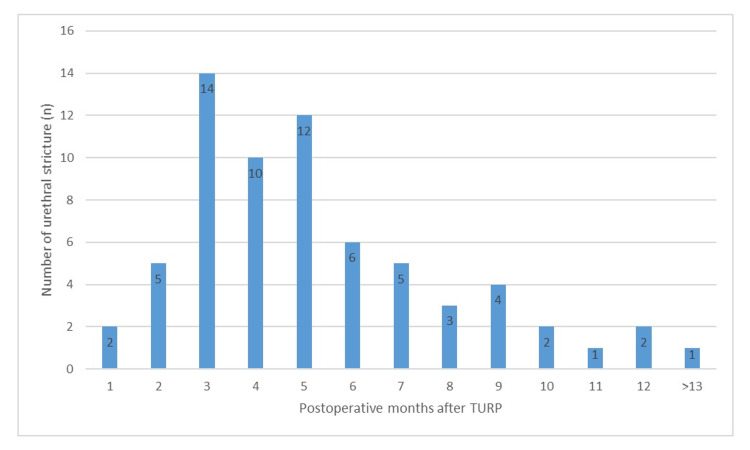
Time to the development of urethral stricture

In the analysis of time to the development of urethral stricture in patients with urethral stricture, it was revealed that stricture most commonly occurred in the third (20.9%) and fifth months (17.9%) postoperatively and that only one patient developed urethral stricture after 12 months postoperatively (Figure 1).

## Discussion

Despite a century-long period after the first TURP surgery, urethral stricture remains an important complication of TURP surgery [10]. The present study examined the long-term outcomes of cases of urethral stricture that developed after TURP surgery in a single center. The results indicated that urethral stricture is likely to develop in approximately 4% of patients after TURP at the end of a minimum of a 12-month follow-up period. In addition, logistic regression analysis showed that prolonged operative time and the number of comorbidities were effective factors in the development of urethral stricture after TURP.

Literature indicates that prolonged operation time has a significant role in the etiopathogenesis of urethral stricture after TURP [10]. Mundy et al. suggested that the use of prolonged resectoscope results in subepithelial fibrosis by causing urethral inflammation and ischemia in the urethral mucosa, thereby leading to mucosal damage and increasing the risk of urethral stricture after TURP [11]. Komura et al. evaluated 136 patients and reported that prolonged operative time increases the risk of developing urethral stricture [12]. A 2015 study evaluated 110 patients and reported that the risk of urethral stricture increased significantly after TURP surgeries that lasted more than 60 minutes [8]. Similarly, Tao et al. reported that a slow resection rate (i.e. prolonged operative time) increased the risk of developing urethral stricture [13]. In our study, operative time was longer in patients with urethral stricture than in patients without urethral stricture, and prolonged resectoscope time was an effective factor in the development of urethral stricture.

Diabetes mellitus (DM) is the most well-known comorbidity associated with urethral stricture occurring after TURP [8-9]. Similarly, Grechenkov et al. noted that the presence of DM is an important risk factor for urethral stricture [8] and Kumsar et al. reported that poor glycemic control increases the risk of developing urethral stricture in the early postoperative period [9]. In contrast, our study included patients with other common comorbidities (asthma, COPD, CAD, hypertension) that could cause tissue ischemia and blood supply disorders and indicated that the risk of early urethral stricture after TURP increased as the comorbidity burden of the patient increased.

There are studies reporting that prostate volume is a factor associated with urethral stricture [8,12]. Similarly, in our study, prostate volume, the amount of tissue removed during surgery, and perioperative blood loss were associated with urethral stricture in univariate analysis conducted at the first stage of the study, though no such relationship was found in multivariate regression analysis. These results indicate that although prostate volume, the amount of tissue removed during surgery, and perioperative blood loss are not directly effective factors in the development of urethral stricture; these factors are natural risk factors arising from prolonged operative time.

Larger resectoscope diameter has been reported to be among the factors increasing the risk of urethral stricture after TURP [14-15]. Since the standard 26 Fr resectoscope sheath is routinely used in our clinic, the relationship between resectoscope diameter and stricture could not be evaluated.

In our study, urethral stricture developed in 3.9% of the patients after a minimum of 12 months of follow-up. The reported incidence of urethral stricture after TURP varies between 1.7% and 9.8% [4,15-16]. Similarly, Michielsen et al. reported that urethral stricture developed in 17 (2.9%) out of 594 patients who underwent TURP surgery with the mono-polar system [17]. In a similar way to our findings, Seçkiner et al. and Tefekli et al. reported the incidence of urethral stricture after monopolar TURP as 4.2% and 2.1%, respectively [16,18]. Erturhan et al. reported that urethral stricture developed in 1.7% of 120 patients at the end of the 12-month follow-up period [15]. Although these findings are consistent with those of our study, it should be noted that these studies included relatively small numbers of patients. In contrast, a 2016 study by Tao et al. evaluated a total of 373 patients and reported the rate of urethral stricture as 7.8%. This high rate could be explained by the fact that that study, unlike ours, included patients with intraoperative urethral mucosal injury as well [13].

Our study was limited in several ways. First and foremost, the study had a retrospective design. Although there are studies in the literature reporting on other factors contributing to the development of urethral stricture after TURP, such as electric current leakage, infections, and the temperature of the solution used [7,12,17,19], data on such factors could not be accessed and these potential risk factors could not be analyzed due to the retrospective nature of the study. Second, due to the low number of patients with urethral stricture, comorbidities could not be analyzed separately and a cut-off value could not be determined for operative time. Finally, surgeries were not performed by a single surgeon.

## Conclusions

Urethral stricture is a potential complication that may develop in the early period after TURP surgery in BPH patients. Prolonged operative time and high comorbidity burden are factors that increase the risk of urethral stricture.

## References

[1] Sonmez G, Topaloglu US, Keske M, Demirtas A (2020). Efficacy of alfuzosin in male patients with moderate lower urinary tract symptoms: is metabolic syndrome a factor affecting the outcome?. Urol J.

[2] Herden J, Ebert T, Schlager D (2021). Perioperative outcomes of transurethral resection, open prostatectomy, and laser therapy in the surgical treatment of benign prostatic obstruction: a "Real-world" data analysis from the URO-Cert Prostate Centers. Urol Int.

[3] Bruce A, Krishan A, Sadiq S, Ehsanullah SA, Khashaba S (2021). Safety and efficacy of bipolar transurethral resection of the prostate vs monopolar transurethral resection of prostate in the treatment of moderate-large volume prostatic hyperplasia: a systematic review and meta-analysis. J Endourol.

[4] Rassweiler J, Teber D, Kuntz R, Hofmann R (2006). Complications of transurethral resection of the prostate (TURP)—incidence, management, and prevention. Eur Urol.

[5] Varkarakis J, Bartsch G, Horninger W (2004). Long-term morbidity and mortality of transurethral prostatectomy: a 10-year follow-up. Prostate.

[6] Sun F, Sun X, Shi Q, Zhai Y (2018). Transurethral procedures in the treatment of benign prostatic hyperplasia. A systematic review and meta-analysis of effectiveness and complications. Medicine (Baltimore).

[7] Doluoglu OG, Gokkaya CS, Aktas BK, Oztekin CV, Bulut S, Memis A, Cetinkaya M (2012). Impact of asymptomatic prostatitis on re-operations due to urethral stricture or bladder neck contracture developed after TUR-P. Int Urol Nephrol.

[8] Grechenkov AS, Glybochko PV, Alyaev YG, Bezrukov EA, Vinarov AZ, Butnaru DV, Sukhanov RB (2015). Risk factors for anterior urethral strictures after transurethral resection of benign prostatic hyperplasia [Article in Russian]. Urologiia.

[9] Kumsar S, Sağlam HS, Köse O, Budak S, Adsan O (2014). Relationship between development of urethral stricture after transurethral resection of prostate and glycemic control. Urol Ann.

[10] Wang JW, Man LB (2020). Transurethral resection of the prostate stricture management. Asian J Androl.

[11] Mundy AR, Andrich DE (2011). Urethral strictures. BJU Int.

[12] Komura K, Inamoto T, Takai T (2015). Incidence of urethral stricture after bipolar transurethral resection of the prostate using TURis: results from a randomised trial. BJU Int.

[13] Tao H, Jiang YY, Jun Q, Ding X, Jian DL, Jie D, Ping ZY (2016). Analysis of risk factors leading to postoperative urethral stricture and bladder neck contracture following transurethral resection of prostate. Int Braz J Urol.

[14] Günes M, Keles MO, Kaya C (2015). Does resectoscope size play a role in formation of urethral stricture following transurethral prostate resection?. Int Braz J Urol.

[15] Erturhan S, Erbagci A, Seckiner I, Yagci F, Ustun A (2007). Plasmakinetic resection of the prostate versus standard transurethral resection of the prostate: a prospective randomized trial with 1-year follow-up. Prostate Cancer Prostatic Dis.

[16] Tefekli A, Muslumanoglu AY, Baykal M, Binbay M, Tas A, Altunrende F (2005). A hybrid technique using bipolar energy in transurethral prostate surgery: a prospective, randomized comparison. J Urol.

[17] Michielsen DP, Coomans D (2010). Urethral strictures and bipolar transurethral resection in saline of the prostate: fact or fiction?. J Endourol.

[18] Seckiner I, Yesilli C, Akduman B, Altan K, Mungan NA (2006). A prospective randomized study for comparing bipolar plasmakinetic resection of the prostate with standard TURP. Urol Int.

[19] Park JK, Lee SK, Han SH, Kim SD, Choi KS, Kim MK (2009). Is warm temperature necessary to prevent urethral stricture in combined transurethral resection and vaporization of prostate?. Urology.

